# Mesopelagic microbial carbon production correlates with diversity across different marine particle fractions

**DOI:** 10.1038/s41396-020-00880-z

**Published:** 2021-01-15

**Authors:** Chloé M. J. Baumas, Frédéric A. C. Le Moigne, Marc Garel, Nagib Bhairy, Sophie Guasco, Virginie Riou, Fabrice Armougom, Hans-Peter Grossart, Christian Tamburini

**Affiliations:** 1 Aix-Marseille Université, Université de Toulon, CNRS, IRD, Mediterranean Institute of Oceanography (MIO, UM 110), Marseille, France; 2 Department of Experimental Limnology, Leibniz Institute of Freshwater Ecology and Inland Fisheries, Stechlin, Germany; 3 Institute of Biochemistry and Biology, Postdam University, 14469 Potsdam, Germany

**Keywords:** Microbial ecology, Biogeochemistry

## Abstract

The vertical flux of marine snow particles significantly reduces atmospheric carbon dioxide concentration. In the mesopelagic zone, a large proportion of the organic carbon carried by sinking particles dissipates thereby escaping long term sequestration. Particle associated prokaryotes are largely responsible for such organic carbon loss. However, links between this important ecosystem flux and ecological processes such as community development of prokaryotes on different particle fractions (sinking vs. non-sinking) are yet virtually unknown. This prevents accurate predictions of mesopelagic organic carbon loss in response to changing ocean dynamics. Using combined measurements of prokaryotic heterotrophic production rates and species richness in the North Atlantic, we reveal that carbon loss rates and associated microbial richness are drastically different with particle fractions. Our results demonstrate a strong negative correlation between prokaryotic carbon losses and species richness. Such a trend may be related to prokaryotes detaching from fast-sinking particles constantly enriching non-sinking associated communities in the mesopelagic zone. Existing global scale data suggest this negative correlation is a widespread feature of mesopelagic microbes.

## Introduction

The oceanic biological carbon pump (BCP) is a complex set of mechanisms that regulates Earth’s carbon (C) cycle by sequestrating part of the photosynthetically fixed carbon dioxide (CO_2_) in the deep ocean (>1000 m) and the seafloor [1]. In essence, particulate organic carbon (POC) is transported into the ocean’s interior via gravitational sinking of marine particles [2, 3] of various types and sinking rates. The depth at which marine particles are remineralized in the ocean exerts a strong influence on the CO_2_ air/sea balance. The deeper this depth is, the more CO_2_ is stored over a long period of time, locked away from the atmosphere [4]. This CO_2_ sequestration is mostly vectored by gravitational sinking particles [2].

POC downward flux and sequestration (that is POC flux below 1000 m) in the deep ocean is controlled by photosynthetic primary producers, but also by heterotrophic organisms transforming POC during its descent into the mesopelagic zone (euphotic zone base to 1000 m). During their sinking, particles are grazed/repackaged into dense fecal pellets and/or fragmented into smaller particles by zooplankton [5] and abiotic processes [6]. Their organic carbon content is mainly remineralized by heterotrophic microorganisms [7, 8]. Hence, the POC flux is attenuated with depth through zooplankton activity and abiotic processes, but the final remineralization step (releasing dissolved CO_2_) is mainly performed by heterotrophic microorganisms [9, 10]. However, currently no consensus exists either on the environmental drivers or the ecological processes that may dictate the activity of the heterotrophic prokaryotic community associated with fast-sinking particles at increasing depth. This prevents any adequate mechanistic representation of the activity, community network, or ecological lifestyle of particle-associated prokaryotes in oceanic food webs and biogeochemical models [10, 11].

Previoulsy, the majority of studies on prokaryotes attached to particles used conventional seawater bottle samplers followed by size fractionated filtration [12]. However, such samplers do not actively and quantitatively collect intact gravitational sinking particles [13, 14] and an adequate harvest of sinking particles remains a challenge [15]. Studies have also looked at the diversity of sinking particles collected using sediment traps [16], but did not relate prokaryotic production measurements nor estimates to it. Therefore, distinction between gravitational sinking particles vs. other particles (sinking or non-sinking fractions, see definition below), and their associated biogeochemical rates and/or ecological features has been rarely addressed.

For the first time, Turley and Mackie [17] used the Marine Snow Catcher (MSC [18]) to specifically sample different particle fractions to investigate in essence, fast-sinking vs. non-sinking particles, and their attached prokaryotic cells concentration and activities. They found inconsistencies between the intrinsic carbon demand of the prokaryotic community and the corresponding carbon supply in the upper mesopelagic zone with prokaryotic heterotrophic production (PHP) being lower on fast-sinking particles relative to non-sinking ones. This is due to the scarcity of fast-sinking particles in oceanic waters. Later, Duret et al. [19] segregated particles using the MSC to study prokaryotic diversity associated with sinking vs. non-sinking particle fractions. Using 16 S rRNA gene sequencing, they revealed a strikingly different ecological behavior with *K*-strategists (specialists) prokaryotes thriving on the non-sinking fraction and *r*-strategists (generalists) being observed in high proportions on the sinking particles. This highlights important progresses in our mechanistic understanding of mesopelagic community dynamics on different particle fractions, but from a biogeochemical perspective, our understanding of the ecological drivers of C cycling within the mesopelagic is still limited.

Recently, a positive linear correlation was found between prokaryotic species richness and associated heterotrophic productivity in freshwaters [20]. Yet no study has related remineralization rates to the diversity of prokaryote communities in the ocean’s interior. Such trait-based approaches are being increasingly used in marine ecology with plankton communities having lately received attention on a global scale [21], in particular primary producers [22]. However, the mesopelagic prokaryotic community and more specifically its particle associated fractions have received much less attention despite their known biogeochemical and climatic importance [23]. To date, concepts in ecological processes such as the relationships between secondary production and community diversity remain poorly studied.

Here, we provide the first assessment of remineralization rates of heterotrophic prokaryotes coupled to their diversity associated to the non-sinking and fast-sinking fractions at four depths horizons of the mesopelagic zone in the North Atlantic Ocean. Thereby, the “non-sinking fraction” comprises free-living prokaryotes and those attached to suspended particles, and the “fast-sinking fraction” refers to prokaryotes attached to fast-sinking particles (i.e., gravitational sinking particles) retrieved from the MSC. Measured PHP was used as an indicator of remineralization rates on fast-sinking and non-sinking prokaryotic fractions. Using 16 S rRNA sequencing, we identified key prokaryotic players and their potential ecological and metabolic functions to further predict prokaryotic processes involved in mesopelagic particle remineralization. In this study, we coupled cell-specific PHP rates (and further estimates of prokaryotic C loss rates) to prokaryotic species richness. Our results show that cell-specific rates vary little with depth on fast-sinking particles while they decrease strongly on non-sinking particles. Interestingly, microbial species richness decreases with depth for the fast-sinking particles while increases for non-sinking particles despite energetic resources being potentially available in fast-sinking particles. Finally, we found a strong negative relationship between cell-specific PHP rates and species richness. While the underlying mechanisms driving such a pattern are still unclear, our results suggest this negative correlation may be a widespread ecological trait of mesopelagic prokaryotic communities. This potentially increases C sequestration as the ability of the fast-sinking particle prokaryotic community to degrade organic C may not be as efficient as the ability of the more diverse community observed in the non-sinking fraction at any depths.

## Results and discussion

### Fractions and associated prokaryotic heterotrophic production rates

Using a marine snow catcher (MSC, see reference [18], Fig. S1), we collected and segregated the non-sinking (suspended particle attached prokaryotes and free-living prokaryotes, hereafter “non-sinking,” obtained from the MSC’s top part or Niskin bottles, see methods and supplementary material) and fast-sinking (i.e., gravitational sinking particles collected in the MSC’s plate, see methods and supplementary material) prokaryotic fractions. Samples were taken at 28, 70, 128, and 500 m depth at the Porcupine Abyssal Plain site (PAP site), a longstanding deep observation station in the North Atlantic [24] (see methods for a brief description of basic biogeochemical settings at this location). These depths correspond to MSCs deployed at 10 m, 50 m and 110 m below the base of the mixed layer depth (MLD), i.e., MLD + 10 m, MLD + 50 m, MLD + 110 m, respectively (see reference [25] for details). Conventional bottle samplers (NISKIN bottles) were also used for a direct comparison (see methods for detailed explanation).

On site, vertical profiles of temperature, light (as percentage of photosynthetically available radiation, PAR) and chlorophyll concentrations corresponding to the four MSC deployments were consistent (Fig. S2). Temperature ranged from 15 °C in surface to 12 °C below 100 m depth. Chlorophyll concentrations ranged from 0.5 to 1.7 mg m^−3^ in surface waters and sharply decreased below 50 m depth (Fig. S2). The dominant component of fast-sinking particles were phytodetrital aggregates (96% of sinking POC at 36 m, increasing to 66% at 500 m [25]). The proportion of zooplankton fecal pellets added to unidentified phytodetritus increased with depth but remained below 40% at 500 m [25].

**Fig. 1:**
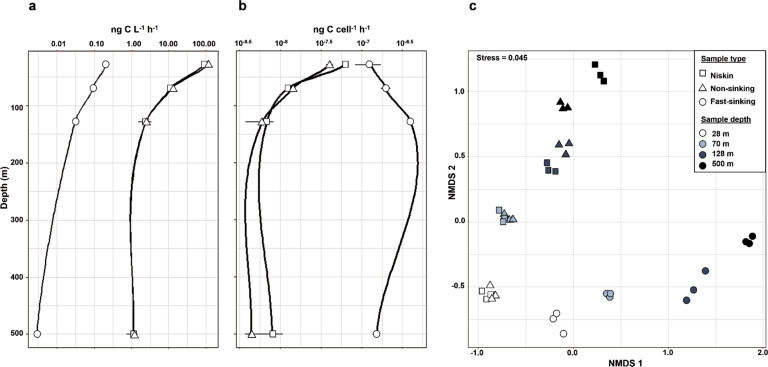
Prokaryotic heterotrophic production and OTUs dissimilarity in the three different fractions. Depth profiles of total (**a**) and cell-specific (**b**) prokaryotic heterotrophic production (PHP) in three different fractions (see main text): (i) seawater sampled with Niskin bottles and (ii) non-sinking and (iii) fast-sinking prokaryotic fractions sampled using a marine snow catcher (MSC) at the PAP site during the DY032 cruise. **c** Non-metric multi-dimensional scaling (NMDS) ordination plot (Bray Curtis distance) showing the dissimilarity of operational taxonomic unit (OTUs) between the different sample types at 28, 70, 128, and 500 m. The stress value of the NMDS analysis is 0.045.

From 28 to 500 m depth, prokaryotic heterotrophic production rates (PHP, i.e., quantification of ^3^H-Leucine uptake by heterotrophic prokaryotes for biomass production) on the non-sinking fraction decreased from 111.755 to 1.161 ng C L^−1^ h^−1^ (Fig. 1a and Table S1). PHP rates on fast-sinking particles decreased by two orders of magnitude, from 0.211 to 0.003 ng C L^−1^ h^−1^ between 28 and 500 m depth when reported to the volume sampled (see methods and supplementary material for details). Normalizing these rates to the number of prokaryotic cells, cell-specific PHP rates (see methods and supplementary material for details) on the non-sinking fraction decreased from 4.1 × 10^−8^ to 4.4 × 10^−9^ ng C cell^−1^ h^−1^ between 28 and 500 m depth (Fig. 1b and Table S1), similarly to those obtained previously [17, 26]. In contrast, cell-specific PHP rates on fast-sinking particles varied little with depth (from 1.29 × 10^−7^ ng C cell^−1^ h^−1^ at 28 m to 1.53 × 10^−7^ ng C cell^−1^ h^−1^ at 500 m with a maximum of 4.00 × 10^−7^ ng C cell^−1^ h^−1^ at 128 m, Fig. 1b). At each sampling depth, cell-specific PHP rates on fast-sinking particles were higher than of the non-sinking fraction (Fig. 1b, Table S1).

Using 16 S rRNA sequences (16 S rRNA are reverse transcripted to cDNA targeting viable prokaryotes), we further assessed dissimilarities (based on the Bray-Curtis dissimilarity index, see further explanation in the methods section) between the different prokaryotic fractions considered. We generated a non-parametric multidimensional scaling (NMDS) ordination based on Bray-Curtis distance of Operational Taxonomic Units (OTUs) distribution in the different fractions (Fig. 1c). Similar to PHP rates, this revealed a clear dichotomy between prokaryotic communities attached to fast-sinking particles (NMDS1) and those belonging to the non-sinking fraction (NMDS2). In addition, dissimilarities between both prokaryotic communities (non-sinking and fast-sinking fractions) increased with depth (Fig. 1c).

**Fig. 2:**
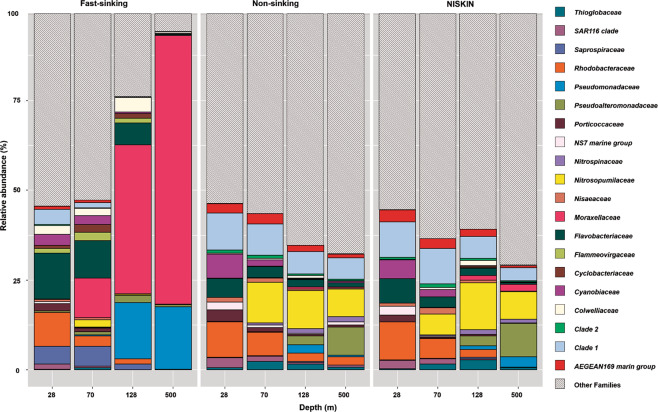
Relative abundance of prokaryotic groups (family level). Three different types of samples were distinguished: (i) seawater sampled with Niskin bottles, (ii) non-sinking and (iii) fast-sinking prokaryotic fractions sampled with a marine snow catcher (MSC) at the PAP site during the DY032 cruise at 28, 70, 128, and 500 m depth. Only the 20 most abundant families are shown, others family proportions are merged into the hatched part.

Our results suggest that prokaryotic diversity and activity (i.e., PHP) differed in two different, but consistent, manners depending on the type of the prokaryotic fraction (Figs. 1 and 2). In addition, given the similarities in activity and diversity between Niskin and MSC’s top samples (both non-sinking and free-living prokaryotes), hereafter non-sinking fraction (see Supplementary Material), we assume that further distinction between Niskin and MSC’s top samples is no longer necessary. This is consistent with previous work showing that bottle samplers avoid fast-sinking particles by design [13, 15] and do not represent bulk activity or diversity as generally assumed [12].

### Diversity of different prokaryotic fractions

The 16S rRNA sequences enabled examination of viable prokaryotic species richness associated with both fast- and non-sinking prokaryotic fractions. The observed species richness (see methods and supplementary material) of prokaryotic communities attached to fast-sinking particles decreased drastically (from 314 to 128) between 28 and 500 m depth. Conversely, species richness increased from 262 to 531 for the non-sinking fraction between 28 and 500 m (Fig. S3 and methods). A similar depth-related increase in species richness of the prokaryotic community of non-sinking prokaryotic fractions has been reported previously for the Atlantic Ocean [27, 28].

The relative abundances of the twenty most represented, active families across all samples are shown in Fig. 2. Results revealed a clear divergence of prokaryotic communities between fast-sinking vs. non-sinking prokaryotic fractions as a function of depth. At 28 m depth, the proportions of the twenty most represented families were relatively similar in both fractions; only their relative abundances differed. The non-sinking prokaryotic community was mainly dominated by *Rhodobacteraceae* and *SAR11 Clade 1* (10% of the relative abundance each) as well as *Flavobacteriaceae* (6%), and the prokaryotic community attached to fast-sinking particles was dominated by *Flavobacteriaceae*, *Rhodobacteraceae*, *Saprospiraceae* and the *SAR11 Clade 1* (13, 9, 5, 4% respectively) (Fig. 2).

At 70 m depth (upper mesopelagic zone [25]), the non-sinking prokaryotic communities changed towards a higher proportion of *Nitrosopumilaceae* and lower proportions of the *SAR11 Clade 1*, *Rhodobacteraceae* and *Flavobacteriaceae* (9, 9, 6, 3% respectively) relative to 28 m. On the fast-sinking particles however, *Flavobacteriaceae* represented 10% while *Moraxellaceae* increased to 11%. The relative abundance of *Rhodobacteraceae* at 70 m depth decreased more in the fast-sinking than in the non-sinking prokaryotic fraction (3 and 6% respectively). In addition, our results highlight the presence of copiotrophs in the fast-sinking fraction (i.e., *Flavobacteraceae* and *Saprospiraceae*). At 128 m, we observed higher proportions of *Nitrosopumilaceae* and lower proportions of *SAR11 Clade 1* (12 and 6%, respectively) in the non-sinking fraction. In this fraction, the relative abundance of *Rhodobacteraceae* was low (2%), yet *Pseudoalteromonadaceae* (marginal at 28 and 70 m, <0.1%), increased to 3% of the total relative abundance at 128 m. At 500 m, *Pseudoalteromonadaceae* along with *Nitrosopumilaceae* dominated the non-sinking fraction (9 and 8%, respectively). Note that over 50% of the non-sinking prokaryotic community consists of low-abundant families (labeled as other families in Fig. 2, <1%). This proportion increased with depth along with an increase of species richness (Fig. S3). Such increase in species richness may indicate that resources are shared between prokaryotes of different origins [29]. In contrast, the fast-sinking prokaryotic communities showed a radically different pattern at depth (Fig. 2). Interestingly, the proportion of *Moraxellaceae* and *Pseudomonadaceae* increased between 128 m (41 and 16%, respectively) and 500 m (75 and 17%, respectively). This dominance resulted in a drastic decline in species richness of the fast-sinking prokaryotic community with depth (Fig. S3). At 500 m depth, 92% of the total community was represented by only two bacterial genera, namely *Acinetobacter* (*Moraxellaceae*) and *Pseudomonas* (*Pseudomonadaceae*), mostly represented by two OTUs each. Among these four OTUs, we assigned with no ambiguity only one OTU with 100% of sequence identity and coverage with *Acinetobacter albensis* ANC 4874 (described by reference [30]**)**. Previous studies found *Acinetobacter* and *Pseudomonas* in water samples collected using Niskin bottles [31, 32], but also on particles collected using sediment traps [33]. *Acinetobacter* is one of the most frequently bacterial lineage retrieved from deep subsurface sediments and *Pseudomonas* genera were previously isolated from deep sediments [34]. The above studies do not correspond to fast-sinking particles only, but this notion suggests that the presence of both *Acinetobacter* and *Pseudomonas* genera associated with marine particles is not unusual.

Differences between fast- and non-sinking prokaryotic communities greatly increased in the mesopelagic zone relative to those in the euphotic zone (Figs. 1 and 2). Between 28 and 500 m depth, environmental conditions drastically differ in terms of light, temperature, and pressure (Fig. S2). Moreover, the quantity [25], and most likely the quality, of available organic carbon [35, 36] also differed for each of the prokaryotic fractions. Organic matter availability was presumably higher on fast-sinking particles as indicated by the presence of copiotrophs. Shifts towards a more diverse non-sinking prokaryotic community with depth were already reported by others [12, 37, 38] and the high prokaryotic species richness observed on non-sinking particles (Fig. S3), whether abundant or scarce, suggests a higher functional potential. This may indicate a higher degree of adaptation of non-sinking relative to fast-sinking porkaryotic communities via mechanisms such as competition and resistance to nutrient scarcity and pressure when changes in environmental conditions occur more slowly [39].

Clear differences in observed PHP rates and community level diversity between both fast- and non-sinking prokaryotic communities raise fundamental questions about the connectivity and dynamics shaping prokaryotic communities in the mesopelagic zone. Previous experimental and theoretical studies demonstrated that plumes of dissolved organic matter forming in the wake of sinking particles do attract free-living, chemotactic prokaryotes [8, 40]. Generally, this is assumed to be the main pathway of sinking particle colonization [41]. In the following section, we challenge this view.

### Dynamics between fast-sinking and non-sinking prokaryotic communities

In the euphotic zone (28 m here), non- and fast-sinking prokaryotic communities share ca. 53% of all OTUs (Fig. S4). In this layer, the combination of biotic and abiotic processes represents a highly interconnected network involving prokaryotic activities, zooplankton feeding and turbulent mixing [9, 19, 42]. As a result, the connectivity between prokaryotic communities associated to the non- and fast-sinking fractions is likely high. Prokaryotes could opt for an attached lifestyle either by active particle colonization (free-living fraction) or by thriving on source particles already before subsequent aggregation to larger particles. This assumption is confirmed by the frequent co-occurrence of most families in the fast- and non-sinking prokaryotic fractions (Fig. 2, 28 m depth). For instance, *Rhodobacteraceae* and *Flavobacteraceae* are known as primary colonizers of particles in the euphotic zone [43].

**Fig. 3:**
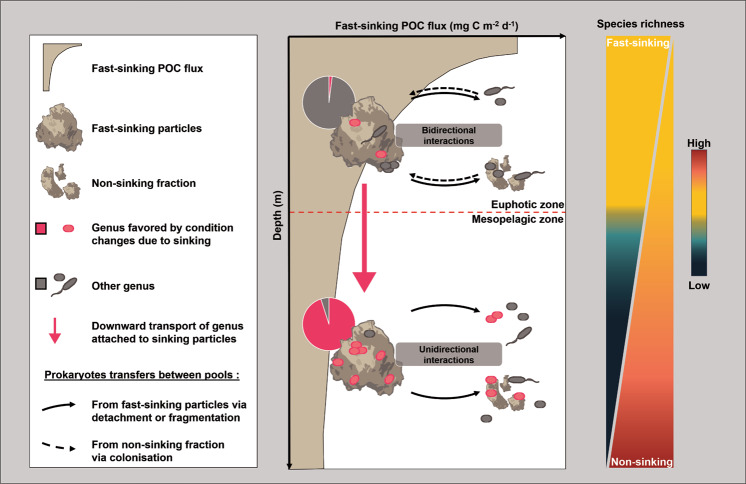
Conceptual diagram illustrating the different interactions between prokaryotic communities associated with fast-sinking and non-sinking fractions. In the euphotic zone, multiple interactions occur in both directions during the initial formation of particles. Dense particles rapidly sink out of the euphotic zone into the mesopelagic zone. The downward transfer of fast-sinking particles is attenuated due to prokaryotic production, but also transports particle-attached prokaryotes from the surface to depth. Changes in environmental conditions induced by sinking (e.g., pressure, temperature, variation in downward flux and substrate quality) result in a drastic decrease in particle-attached species richness favoring certain prokaryotic genera. Further fragmentation of fast-sinking particles into non-sinking particles and cell detachment from particles enriches the surrounding non-sinking prokaryotic communities with these genera. The fast sinking POC flux is plotted after reference [25].

In the mesopelagic zone, drastic changes in community composition and activities occurred among the different prokaryotic fractions (Figs. 1 and 2). From a total of 1013 OTUs, only 16% were still shared by both fast- and non-sinking fractions at 500 m depth (Fig. S4, Table S2). Prokaryotic diversity increased with depth for the non-sinking prokaryotic fraction, but drastically decreased on fast-sinking particles with sequences belonging almost exclusively to two genera, i.e., *Acinetobacter* and *Pseudomonas* (Figs. 2 and 3). Both genera are important for the degradation of organic matter in natural environments [44, 45]. For instance, *Pseudomonas*’ genome is one of the largest in the bacterial world with a battery of genes enabling them to withstand various stresses [46]. Although competitiveness of *Acinetobacte*r and *Pseudomonas* in highly diverse microbial communities is deemed low, they can rapidly adapt to drastic environmental changes in pressure, substrate composition or temperature [44–48]. Consequently, the constant increase in their relative abundance with depth (Fig. 2) could indicate an enhanced selective advantage caused by rapid changes in environmental conditions and substrate composition.

To illustrate this, we estimated growth rates of the total heterotrophic prokaryotic community of cells attached to sinking particles using cell-specific heterotrophic production rates and assuming a conservative conversion factor of 12 fg C cell^−1^ (see “methods” section). These estimates show that the fast-sinking attached heterotrophic prokaryotic community not only persist but is actively growing, varying little with depth from 0.13 to 0.15 h^−1^ at 28 and 500 m depth, respectively, with a maximum growth rate of 0.4 h^−1^ at 128 m. The growth rates of the total heterotrophic prokaryotic community attached to fast-sinking particles are at least one order of magnitude higher than those of the non-sinking fraction. In parallel, while the specific diversity is decreasing on fast-sinking particles, we observed an increase of the relative proportion of two genera with depth (*Acinetobacter* and *Pseudomonas* comprised 57 and 92% of the prokaryotic community at 128 m and 500 m respectively). This, together with the fact that the growth rates of the total heterotrophic prokaryotic community vary little with depth, we propose that the development of both *Acinetobacter* and *Pseudomonas* may explain the observed constancy in the total community growth rates while non-adapted taxa to environmental changes became inactive or disappeared. Interesting enough, testing the effects of hydrostatic pressure and temperature on growth of a piezotolerant *Pseudomonas* experimentally, Kaneko et al. [47] show a constant growth rate until 1000 m depth and a decreasing growth rate from 2000 m depth simulated.

The frequent occurrence of *Acinetobacter* species in deep subsurface sediments [34] together with their capability to produce biofilms and pili as well as their lack of motility and chemotaxis [44] indicate a potential specialization for an attached life style, e.g., on fast-sinking particles. Our results show that *Acinetobacter* and *Pseudomonas* species indeed contribute to 4 and 2%, respectively, of the non-sinking prokaryotic communities at 500 m. Thus, we believe that there is active growth resulting in an active release of prokaryotes rather than an active colonization from the surrounding water during particle sinking. While both, active release of prokaryotes to the surrounding water and active colonization from the surrounding water can occur simultaneously, in this case, we believe that active release of prokaryotes dominates. This is mainly because we observed a clear decrease in prokaryotic diversity on the fast-sinking particles fraction (Fig. 3). If a frequent prokaryotic colonization from non-sinking to fast-sinking fractions would occur at depth, prokaryotic diversity should also increase in the fast-sinking fraction with depth (i.e., mimicking the increase in diversity observed in the non-sinking fraction). This is not the case (Figs. 3 and S3). Also, the core microbiome (OTUs shared among all fractions, Fig. S4) between fractions decreases by 65% between 70 and 500 m. In addition, the four *Acinetobacter* and *Pseudomonas* OTUs of interest (virtually absent in the non-sinking fraction at 28 and 70 m depth) show potential traits of a particle attached lifestyle [44, 49].

The consistent increase of *Acinetobacter* OTUs (all non-motile [50]) in the non-sinking fraction with depth indicates a certain degree of constant prokaryotic detachment from fast-sinking particles. In surface waters, the four *Acinetobacter* and *Pseudomonas* OTUs of interest are present in relatively limited abundance in the fast-sinking fraction and absent in the non-sinking fraction (Fig. 2). Nonetheless, on the fast-sinking fraction, their relative abundance greatly increased with depth, simultaneously appearing in the non-sinking fraction but in relatively limited abundance. The fact that these four OTUs started appearing in the non-sinking fraction at 128 m suggests that sinking particles may act as vertical vectors increasing the prokaryotic diversity in the ambient water at greater depth. Furthermore, our results indicate that taxa poorly represented in the euphotic zone can be positively selected by a strong environmental selective pressure on fast-sinking particles during their rapid descent. This seems to enrich deep waters, and presumably the seafloor, with particle-specific prokaryotes (Fig. 3).

We show that the high prokaryotic diversity in the non-sinking fraction at depth (Figs. 2 and S3) includes multiple taxa with potential capabilities for motility and chemotaxis. Such capacities may allow non-sinking prokaryotes to sense and track loci of high nutrient concentrations, e.g., within plumes of organic matter behind fast-sinking particles [26, 41]. Using Niskin bottles sampling, previous study [51] detected a suite of motility and adhesion genes in *SAR324 clade* cells. However, in the mesopelagic zone we observed that *SAR324 clade* members were basically absent from the fast-sinking particles but contributed between 4 and 7% of the relative abundance of the non-sinking prokaryotic fraction at the order level. This notion suggests that they may attach to non-sinking rather than to fast-sinking particles as has been previously assumed [41]. This suggestion is also confirmed by other prokaryotic taxa, which do not show any evidence of active colonization of fast-sinking particles from the non-sinking prokaryotic communities in the mesopelagic zone (Fig. 2). Consequently, our results suggest mainly an unidirectional transfer of prokaryotes from fast-sinking to the non-sinking fraction in the mesopelagic zone (Fig. 3). The differences between the particle-associated communities appeared to be due to internal changes in the attached microbial community rather than de novo colonization during the sinking of particles as shown previously [52].

### Prokaryotic diversity to heterotrophic production relationships

Multiple studies have investigated the relationship between species richness and climate relevant ecosystem functions provided by the ocean [53]. The vast majority of studies, however, examined phytoplankton primary producers [54]. To our knowledge, combined measurements of oceanic prokaryotic secondary production (i.e., PHP) and species richness related to different prokaryotic fractions have never been done before.

**Fig. 4:**
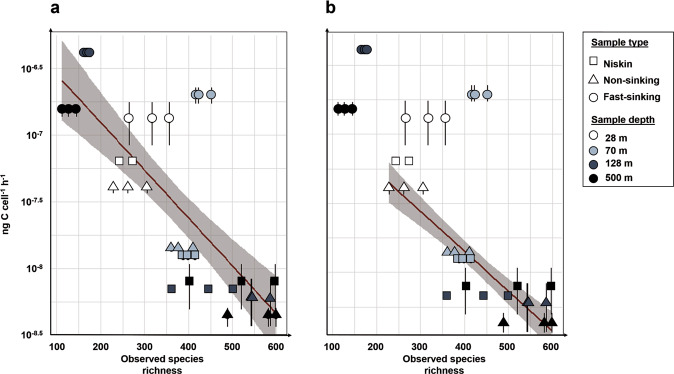
Relationships between species richness and prokaryotic heterotrophic production. Observed species richness (see methods) and cell-specific prokaryotic heterotrophic production (PHP) rates in C units in three different fractions: (i) seawater sampled with Niskin bottles, (ii) non-sinking and (iii) fast-sinking prokaryotic fractions sampled with a marine snow catcher (MSC) at 4 water depths (28, 70, 128, and 500 m). Errors bars represent the standard deviation of PHP rates. Equations of both correlations are provided, see main text for more information. **a** Linear regression including all points (cell-specific PHP = −6.19 –0.00359 x Obs, *r*² = 0.59, *P* = 3.9 × 10^−8^, *n* = 36), (**b**) with points corresponding to fast-sinking particles excluded from the regression (cell-specific PHP = −6.88–0.00289 x Obs, *r*² = 0.80, *P* = 4.3 × 10^−9^, *n* = 24).

We found a strong negative linear correlation between cell-specific PHP rates and observed species richness (see methods and supplementary text) (cell-specific PHP = −6.19 –0.00359 x Obs, *r*² = 0.59, *P* = 3.9 × 10^−8^, *n* = 36; Fig. 4a). When excluding the fast-sinking fraction, the significance of this relationship increased (cell-specific PHP = −6.88 – 0.00289 x Obs, *r*² = 0.80, *P* = 4.3 × 10^−9^, *n* = 24; Fig. 4b). This highlights the fact that particle fractions, dynamics and prokaryotic lifestyle represent important drivers for mesopelagic POC remineralization.

PHP, however, represents only part of the overall prokaryotic C loss (e.g., remineralization). Yet, it is possible that prokaryotic C losses can be estimated using a specific value of prokaryotic growth efficiency [55] (i.e., the amount of new bacterial biomass produced per unit of organic C substrate assimilated). To convert PHP rates into C remineralization rates, we used a prokaryotic growth efficiency of 8% [9] for the non-sinking fraction and a respiratory coefficient of 1 based on respiration measurements by [25] for the fast-sinking fraction (see Table S1 and supplementary text for explanation). This conversion provides confidence that the negative trend between cell-specific PHP rates and species richness (Fig. 4) also holds true for mesopelagic organic C remineralization rates and species richness.

Positive linear relationships between species richness and primary production have been frequently observed in various ecosystems [53, 54]. They can be explained by two theories, whereby an increasing species number initially present in a system could (1) enhance the encounter probability of exceptionally productive species (“selection probability effect” theory) and/or (2) result in more mixed communities of prokaryotic species with more contrasting ecological functions enabling them to use the limiting resources more efficiently and thus reducing interspecies competition (“complementary effect” theory) [56]. Most of these studies focus exclusively on the effects of biodiversity on primary production [57] patterns with little focus on the ocean. In a shallow freshwater estuarine lake, a recent similar study has been performed, but only with surface samples [20]. It revealed a positive linear relationship between prokaryotic species richness and secondary production. In contrast to our study, Schmidt et al. [20] took seasonal variations into account and only distinguished between free-living and particle-attached prokaryotic fractions. Most importantly, no depth-related effects were addressed, which may explain the opposite relationship from the one we have unveiled.

The negative relationship between prokaryotic species richness and secondary production in our study seems to be mainly driven by the non-sinking prokaryotic fraction, which shows a relatively high species richness in combination with low cell-specific PHP rates. In contrast, fast-sinking particles possess relatively low prokaryotic species richness in combination with high cell-specific PHP rates (Fig. 4a). Consequently, high species richness does not necessarily result in high secondary production rates. This relationship is fundamentally different to what is currently accepted for phytoplankton and prokaryotic communities [22]. Yet, the underlying mechanisms driving this trend (Fig. 4) remain largely unknown.

In the mesopelagic ocean, fast- and non-sinking organic matter exhibits a diverse pool of organic substrates [58]. This may increase functional and phylogenetic diversity of prokaryotic communities with increasing depths relative to those in surface waters [59]. In addition, mesopelagic free-living and non-sinking attached prokaryotic communities experience prolonged water-mass residence times [60], which allow for more stable environmental conditions and hence prokaryotic niche separation. We propose that dynamics of mesopelagic fast-sinking particles (high sinking velocities, >27 m d^−1^, see “methods” and reference [25]) in combination with rapid changes in environmental conditions favor “local endemism” (Fig. 2) of the associated prokaryotic communities to gravitational fast-sinking particles. Furthermore, fragmentation of fast-sinking into non-sinking particles and active detachment of cells may potentially enrich the surrounding non-sinking prokaryotic fractions increasing their overall species richness (Fig. 3). This may explain the observed negative relationship between species richness and cell-specific biomass production in the non-sinking prokaryotic fraction.

### Linking community diversity to mesopelagic ocean ecosystem services

Overall, our results suggest that trends between prokaryotic species richness and function are not straightforward and require an adequate assessment before any generalizations can be made. Given the intrinsic differences between prokaryotic lifestyles, additional distinguishing features between fast- and non-sinking prokaryotic fractions seem inevitable. Future efforts to gain a mechanistic understanding of the two main ecosystem functions in the mesopelagic zone [23], i.e., CO_2_ sequestration via mainly fast-sinking particles [23] and mesopelagic C consumption via mainly the non-sinking prokaryotic fraction [9] need to take into account these fundamental differences in prokaryotic lifestyle. For better generalization, we compiled a global range of species richness and PHP rates data (available in the literature, but not necessarily measured simultaneously) across the Atlantic, Pacific and Indian Oceans (Table 1). While this compilation is not exhaustive, species richness seems to consistently increase with depth (from the euphotic to the mesopelagic zone). Similarly, cell-specific PHP rates systematically decrease with depth. Although all observations reported in Table 1 mainly look at the non-sinking fraction, depth-related trends are consistent with our results. This suggests that the trends we observed in the North Atlantic at the PAP site (Fig. 4) may be widespread throughout the ocean and reflect genuine ecological traits of mesopelagic microbes.

**Table 1:** Species richness and measured cell-specific prokaryotic heterotrophic production rates at various locations.

	Depth	Species richness	PHP (ng C cell^−1^ h^−1^)	References species richness	References PHP
Atlantic	Euphotic	20–85	5.81 × 10^−10^–6.90 × 10^−06^	[27, 37]	[27, 37]
	Mesopelagic	40–150	4.52 × 10^−11^–3.4 × 10^−07^	[27, 37]	[27, 37]
Pacific	Euphotic	451–1254	1.77 × 10^−08^–2.08 × 10^−08^	[79–81]	[82]
	Mesopelagic	346–1748	2.78 × 10^−08^–3.75 × 10^−08^	[79–81]	[82]
Indian	Euphotic	46–47	2.4 × 10^−08^–3.00 × 10^−07^	[83]	[84, 85]
	Mesopelagic	56–56	1.8 × 10^−09^–1.00 × 10^−07^	[83]	[84, 85]
Indian/Atlantic/Pacific	Euphotic	240–1100	5.48 × 10^−08^–1.67 × 10^−07^	[12]	[12]
	Mesopelagic	250–1000	4.17 × 10^−08^–1.67 × 10^−07^	[12]	[12]

Genomic sequencing approaches are used [21] increasingly enabling large scale screening of marine prokaryotic and eukaryotic (both auto- and heterotrophic) species richness. In parallel, trait-based models [61] are developed with the intention to include genomic information in conventional biogeochemical models [62]. The links between climate relevant ocean ecosystem functions such as primary production or BCP related fluxes and various ecological traits (e.g., biodiversity or size [21, 54, 63]) are often poorly constrained and occasionally display significant mismatches [64]. Studies on species richness-function relationships are keys to a better mechanistic understanding, required urgently for future projections of major ocean-climate feedback mechanisms.

Furthermore, large scale assessment of mesopelagic prokaryotic communities [22] and their associated metabolic functions are necessary to elucidate the importance of mesopelagic prokaryotic communities for oceanic CO_2_ sequestration [9]. Assuming that functionality changes in accordance to diversity, our study shows a direct relationship between prokaryotic species richness and potential functionality, which seem to be controlled by the rate of changes in environmental conditions during particle sinking. This unique relationship has profound implications for microbial C loss during particle sinking and therefore for the efficiency of the oceanic BCP. We advocate that deciphering the prokaryotic species richness-function relationship is crucial to adequately represent climate relevant biogeochemical gradients. This is required for the future generation of biogeochemistry models based on the ecological traits of different prokaryotic communities (e.g., non- vs. fast-sinking) throughout the oceanic water column.

## Material and methods

### Cruise details

We collected samples during the DY032 cruise (RRS DISCOVERY, NERC, Southampton) from the 20^th^ of June to the 08^th^ of July 2015 at the PAP site (Porcupine Abyssal Plain, 49.0°N, 16.5°W) in the North Atlantic. The cruise took place approximately two weeks after the main spring bloom [25]. The depth of the mixing layer (28 m) was determined by reference [25] following methods presented in reference [65].

### Sampling strategy

Samples were collected at 28, 70, 128, and 500 m depth using both Niskin bottles and a MSC [18]. These depths correspond to MSCs sampling at 10 m, 50 m, and 110 m below the base of the MLD, i.e., MLD + 10 m, MLD + 50 m, MLD + 110 m, respectively [25]. The MSC is designed to reduce turbulence during sampling in such manner that the particles harvested are not biologically, chemically, or morphologically altered. After sampling, MSCs were left 2 h on deck allowing particles to settle. Fast-sinking particles were thereby deposited at the bottom of the MSC and collected in a dedicated tray, slow-sinking particles in the 7-litre compartment above the plate and non-sinking fraction in the upper part of the MSC [18]. Three types of samples are shown in this study: seawater and suspended particles in either Niskin^®^ bottles or the MSC’s top part (both termed non-sinking), and fast-sinking particles from the MSC’s plate (Fig. S1). The entire volume of the plate (around 243 mL) was sampled with a pipette to collect all fast-sinking particles. As fast-sinking particles were concentrated during a 2 h period of settlement, measurements values required normalization to the entire volume of the MSC (100 L). In addition, we assumed that the fast-sinking fraction contained seawater, slow-sinking, suspended and primarily fast-sinking particles. We therefore use the formula shown in Fig. S1 to obtain values associated to fast-sinking particles only, quantitatively removing the contribution of other fractions. This formula is adapted from reference [18], who used it for the slow-sinking fraction (a fraction not considered in the present study). On occasion, the fast-sinking particle slurry collected in the plate was further diluted in sterile seawater (filtered and autoclaved) to ease handling. Then, dilution factors were also applied (Fig. S1). Various measurements were performed on the fractions described above. These are detailed below. We estimated the maximum temperature the 100 L of the MSC may reach after 2 h settling on deck to calorimetry and the heat transfer calculations. We used a specific heat capacity of water and the heat conductivity properties of the MSC walls (1.5 m height, 0.3 m radius and 0.01 m thickness of plastic), for an initial temperature of 12 °C (see Fig. S2), a temperature on deck of 18 °C and assuming steady state. After 2 h, the temperature of the water collected will be 14.6 °C.

### Prokaryotic heterotrophic production measurements (PHP)

Prokaryotic heterotrophic production (PHP) was measured, in triplicates, by incorporating L-[4,5-3H]-Leucine, (^3^H-Leu, 108 Ci mmol^−1^ of specific activity, PerkinElmer^®^) to get a final saturation concentration of 20 nM according to [66]. Samples collected at 28, 70, and 128 m were incubated for four hours. Samples collected at 500 m were incubated for 6 h. All samples were incubated at temperatures corresponding to that observed in situ. To convert pmol Leu L^−1^ h^−1^ into produced carbon biomass (equal to carbon loss), we chose a conversion factor of 1.55 kg C pmol^−1^ Leu incorporated and assumed an isotopic dilution equal to 1 according to [67].

### Total community growth rates

We determined growth rates of the total prokaryotic community attached to sinking particles using cell-specific PHP rates (shown in Fig. 1). We assumed a conservative conversion factor of 12 fg C cell^−1^ from [68]. Our calculation is explained by the following dimensional equation (1):

1 $$\left( {ng\;C\;cell^{ - 1}h^{ - 1}} \right)/\left( {\;fg\;C\;cell^{ - 1}} \right) = h^{ - 1}$$

This conversion yields prokaryotic growth rates expressed in h^−1^.

### Heterotrophic prokaryotic cells concentration

Heterotrophic prokaryotic cells concentration from non-sinking and Niskin samples were counted using a flow cytometer “Flow cytometer analyzer FACSCalibur” (BD Biosciences). Ten ml of sample were collected, fixed with 100 µl of 25% glutaraldehyde and stored at −80 °C for further analysis. We combined our cell concentration to that found by reference [17] to estimate the concentration of cells associated with fast-sinking particles. Taking cell concentration of the non-sinking pool at 28, 70, 128, and 500 m depth and power law estimations (at the same depths) derived from their sinking pool values, we were able to obtain ratios between both fractions. Applying these ratios to cell concentration of our non-sinking pool, we provide an estimation of cell concentration associated with fast-sinking particles. Non-sinking samples ranged from 2.10 ± 0.94 × 106 to 1.97 ± 0.99 × 105 cells ml^−1^ from 28 to 500 m, respectively. Briefly, fast-sinking particles would represent a concentration factor between 1768 and 397 at 28 m and between 836 and 377 at 500 m. Thus, the fast-sinking pool from 28 to 500 m would account for 2.27 ± 2.03 × 109 and 1.2 ± 0.64 × 108 prokaryotic cells ml^−1^ of particle, respectively.

### 16S rRNA extraction, sequencing and metabarcoding library preparation

For Niskin, MSC non-sinking and fast-sinking fractions, 1750, 1750, and 10 ml were filtered onto 0.2 µm membrane, respectively. Filters were treated with TE-Lysis buffer (20 mM Tris, 25 mM EDTA, 1 µg µl^−1^ Lysozyme) followed by 10% SDS (sodium dodecylsulfate). Extractions were performed twice with an equal volume of phenol:chloroform:isoamyl alcohol pH 6 16S-rRNA. 16S-rRNA samples were then treated with TurboDNaseTM (Ambion^®^, Thermo Fisher Scientific Corp.) and reverse transcribed into cDNA by RT-PCR using SuperScript^®^ IV Reverse Transcriptase with random primers (Life Technologies, Thermo Fisher Scientific Corp.). For ribosomal diversity analysis, the V4 region of the bacterial and archaeal 16 S cDNA product were amplified using universal primer sets [69], 515F-Y (5′-GTGYCAGCMGCCGCGGTAA-3′) [70], and 806RB (5′-GGACTACNVGGGTWTCTAAT-3′) [71] as well as 2.5 U/50 µL TaKaRa PrimeSTAR^®^ GXL DNA polymerase (OZYME). The 16 S amplicons were sequenced via MiSeq Illumina (paired end 2∗ 250) platform GeT of Genotoul (https://get.genotoul.fr/en/). 16S-rRNA raw read sequences are deposited on Short Read Archive (accession number are ranged from SRR11997098 to SRR11997133 and the SRA accession is PRJNA638895). Blanks were systematically performed and checked on electrophoresis gels. No nucleic acid was amplified on any of our blanks suggesting no contamination during sample processing.

### Analysis of sequencing data

Raw data was analyzed using DADA2, a model-based approach for correcting amplicon sequencing errors [72]. After inspection of quality read profiles, the 16 S rRNA paired-end reads were quality trimmed and only reads >150 bp were retained. The paired-end reads were then dereplicated, denoised (DADA2 error correction model), assembled, and chimeras were discarded (Table S3). The high-quality and denoising sequences obtained are amplicon sequence variants (ASVs). A post clustering curation [73] of ASVs into OTU was applied to minimize the effect of intraspecific heterogeneity of rDNA copy number in strains/species. The OTU clustering is based on a sequence identity threshold of 97% and performed by DECIPHER package [74]. The taxonomic assignment of OTUs was performed using the SILVA_132 database [75] with 100% identity required for species rank. Finally, sub-sampling normalization, alpha and beta diversity were characterized by the Phyloseq, R package [76]. From a total of 1121933 sequences found in our 36 samples, 850234 sequences, i.e., 76% were retained after the pre-processing (quality control, merging and chimera removal, Table S3). In addition, rarefaction curves quickly plateaued after pre-processing (Fig. S5). This verifies a sufficient sequencing depth for our study (Table S3).

Our metabarcoding library has been generated from short 16 S amplicons (about 290 bp). The size of the 16 S amplicons does not allow accurate taxonomic assignment. Yet, such approach does not capture sufficient sequence variation to adequately discriminate closely related taxa [77]. As a result, we assigned only 31% of all OTUs at the genus level in all our samples combined. This low assigned taxonomic fraction can also result from uncharacterized marine microorganisms absent from 16 S databases. In comparison 54% were assigned at the family level. For this reason, we did not assign further than the family level. However, we deepened the phylogenetic assignment only for the few genera that were most relevant (see Fig. 2).

### Statistical analysis

Statistical analyses were performed with the R statistics software (https://www.r-project.org). The species richness (observed species richness (species number in a sample) and Chao1 (the common species richness estimator)), the community diversity (Shannon index) and evenness (Simpson index) were all calculated using the alpha diversity function from the Phyloseq R package [76]. Non-metric multidimensional scaling (NMDS) ordination plot (Fig. 1) of Bray-Curtis community dissimilarities between samples was performed using the Phyloseq R package [76]. The resulting graphs were all constructed using ggplot2 R package [78]. Statistical tests are provided in Table S4 and diversity indices in Fig. S3 and Table S5. All error bars in figures correspond to standard deviations from triplicate measurements.

## Supplementary information

Supplementary material
