# Supplementary material for: Mesopelagic microbial carbon production correlates with diversity across different marine particle fractions
Source: ISME J. 2021 Jan 15;15(6):1695–708. doi: 10.1038/s41396-020-00880-z (PMC8163737; doi:10.1038/s41396-020-00880-z)
Supplement: Supplementary file 1 — Supplementary material [file 41396_2020_880_MOESM1_ESM.pdf]

Supplementary information for Mesopelagic microbial carbon production correlates with diversity across different marine particle fractions by Baumas et al.

Diversity indices

Species richness was determined using the “observed species richness” and the “Chao1” indices, both of which are indicators of species richness (see methods). Both indicate similar results (Figure S2). Therefore, we only show the observed species richness hereafter. We use the observed species richness because it is intercomparable while commonly used Shannon and Simpsons indices (Figure S2) are not.

PHP to prokaryotes rates conversion.

Reference [1] measured total O_2 consumption on fast sinking particles [2]. We normalized these numbers by the amount of fast-sinking particles per volume of water. Further, assuming a respiratory quotient (RQ) of 1 (mol O_2: mol CO_2), we determined C losses presented in table S1. Note that this calculation would yield a prokaryotic growth efficiency (PGE) of 2.3%. For the non-sinking fraction, estimates of C loss rates were determined using PHP rates (Figure 1) and converted by a prokaryotic growth efficiency (PGE) of 8%. 8% is the median of 26 measured PGE into the mesopelagic zone across the North Atlantic ocean [3].

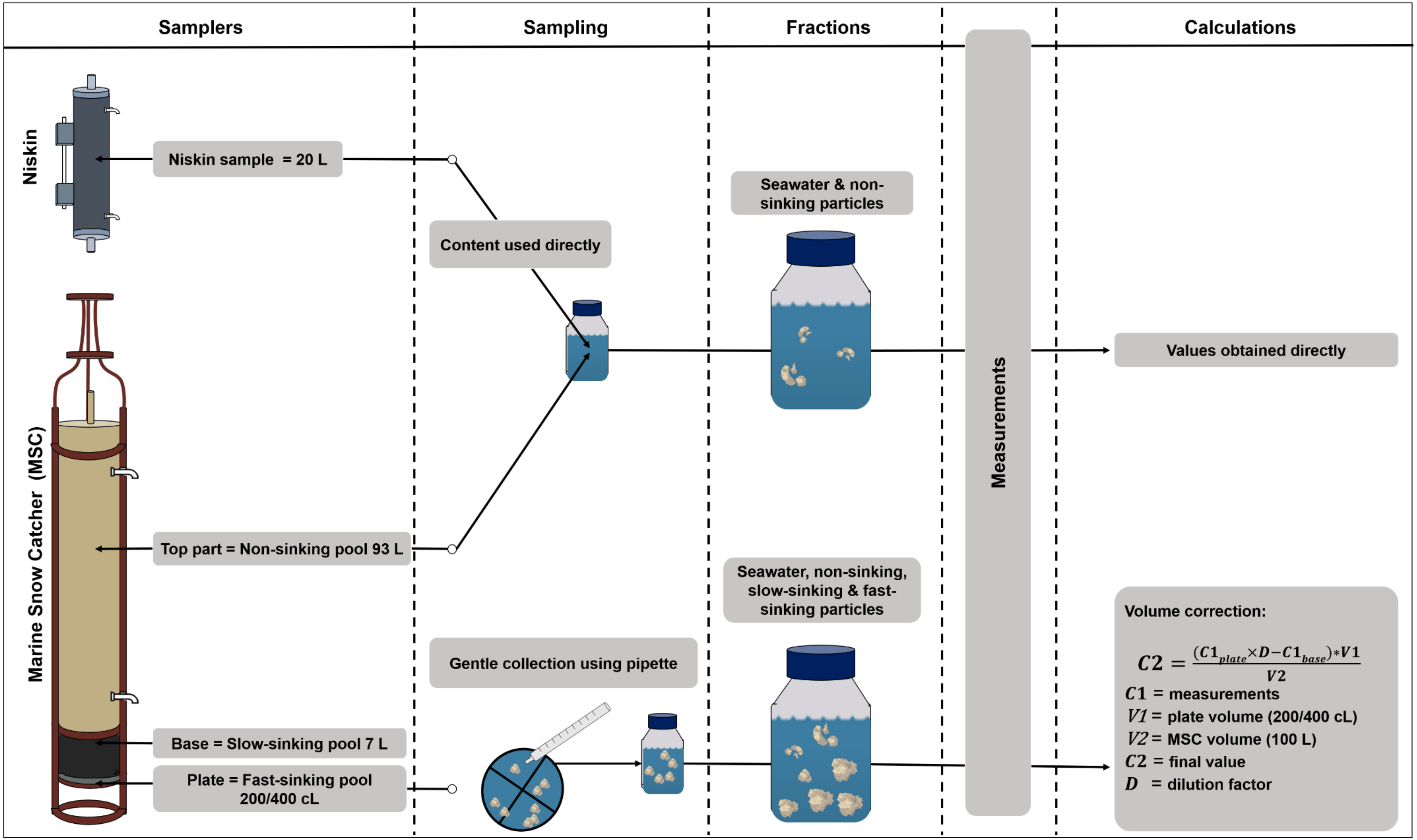

Samplers
Sampling
Fractions
Calculations
Niskin
Niskin sample = 20 L
Content used directly
Seawater & non-sinking particles
Measurements
Values obtained directly
Marine Snow Catcher (MSC)
Top part = Non-sinking pool 93 L
Seawater, non-sinking, slow-sinking & fast-sinking particles
Gentle collection using pipette
Base = Slow-sinking pool 7 L
Plate = Fast-sinking pool 200/400 cL
Volume correction:
$C2 = \frac{(C1_{plate} \times D - C1_{base}) * V1}{V2}$
$C1$ = measurements
$V1$ = plate volume (200/400 cL)
$V2$ = MSC volume (100 L)
$C2$ = final value
D = dilution factor

Figure S1: Sampling strategy. After settling on deck for two hours in the Marine Snow Catcher [4] (MSC),fractions were sampled as follows: the volume required for measurements in the Niskin and non-sinking fractions was used as the sample was considered to be homogeneous. The collection plate at the bottom of the MSC (where fast-sinking particles were concentrated for two hours) were gently pooled together using pipettes. Measurements were then normalized to the entire volume of the MSC (100 L). A correction was necessary to obtain values exclusively related to fast-sinking particles. This formula is adapted from reference [4] who used it for the slow-sinking fraction initially (fraction not considered in the present study). This is because fast-sinking samples contained fast-sinking particles but also slow-sinking as well as non-sinking fraction and seawater (suspended material). When the amount of particles collected was not sufficient, the particle slurry was then further diluted in sterile water (filtered and autoclaved). The dilution factor was then applied to the various measurements.

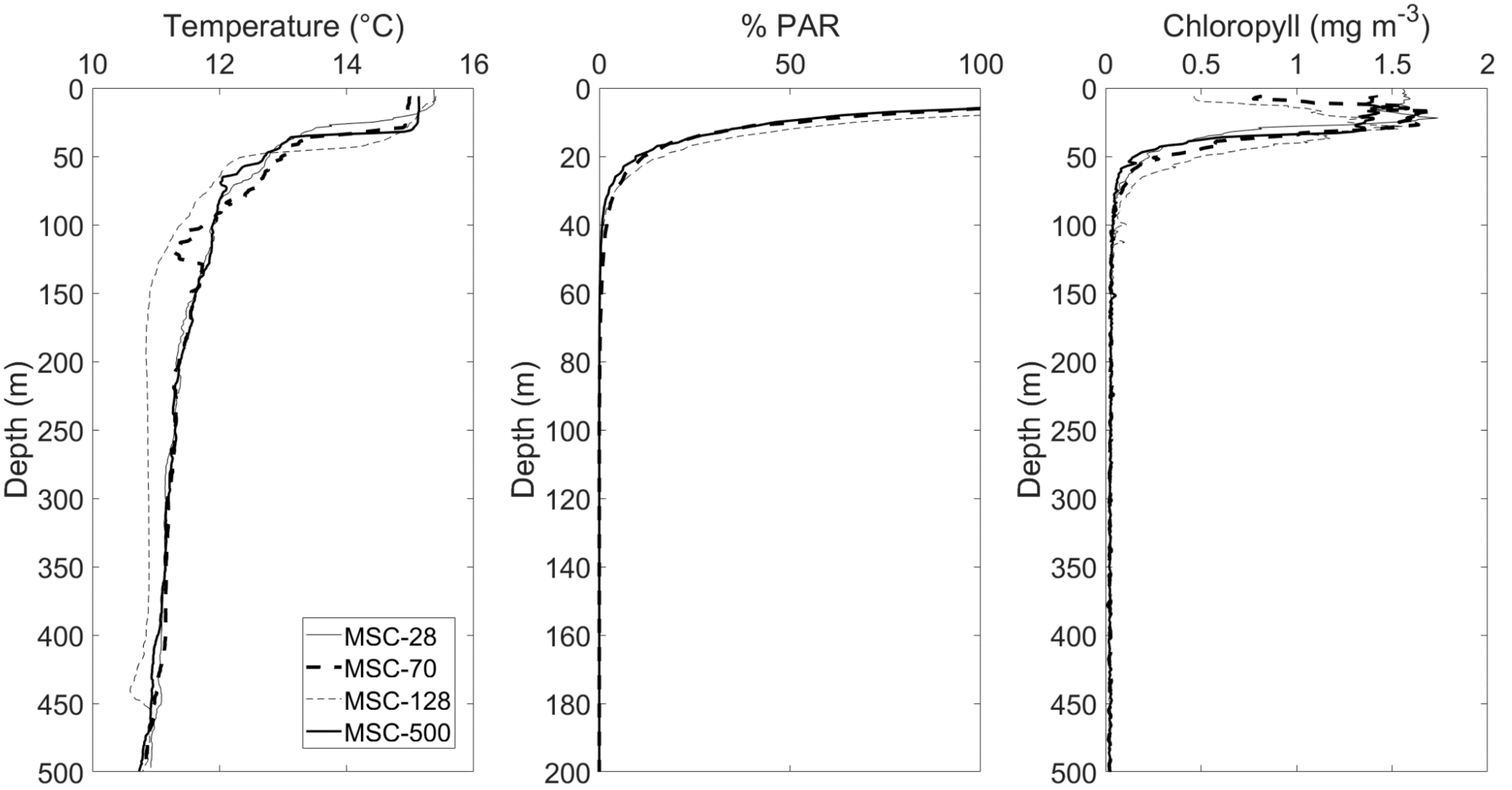

Figure S2: Depth profiles of temperature (°C), percentage of photosynthetically available radiation (%PAR) and Chlorophyll concentration from fluorescence at the four MSC deployments locations. Note that no PAR profile exists for MSC-28 because the corresponding CTD was deployed during nighttime.

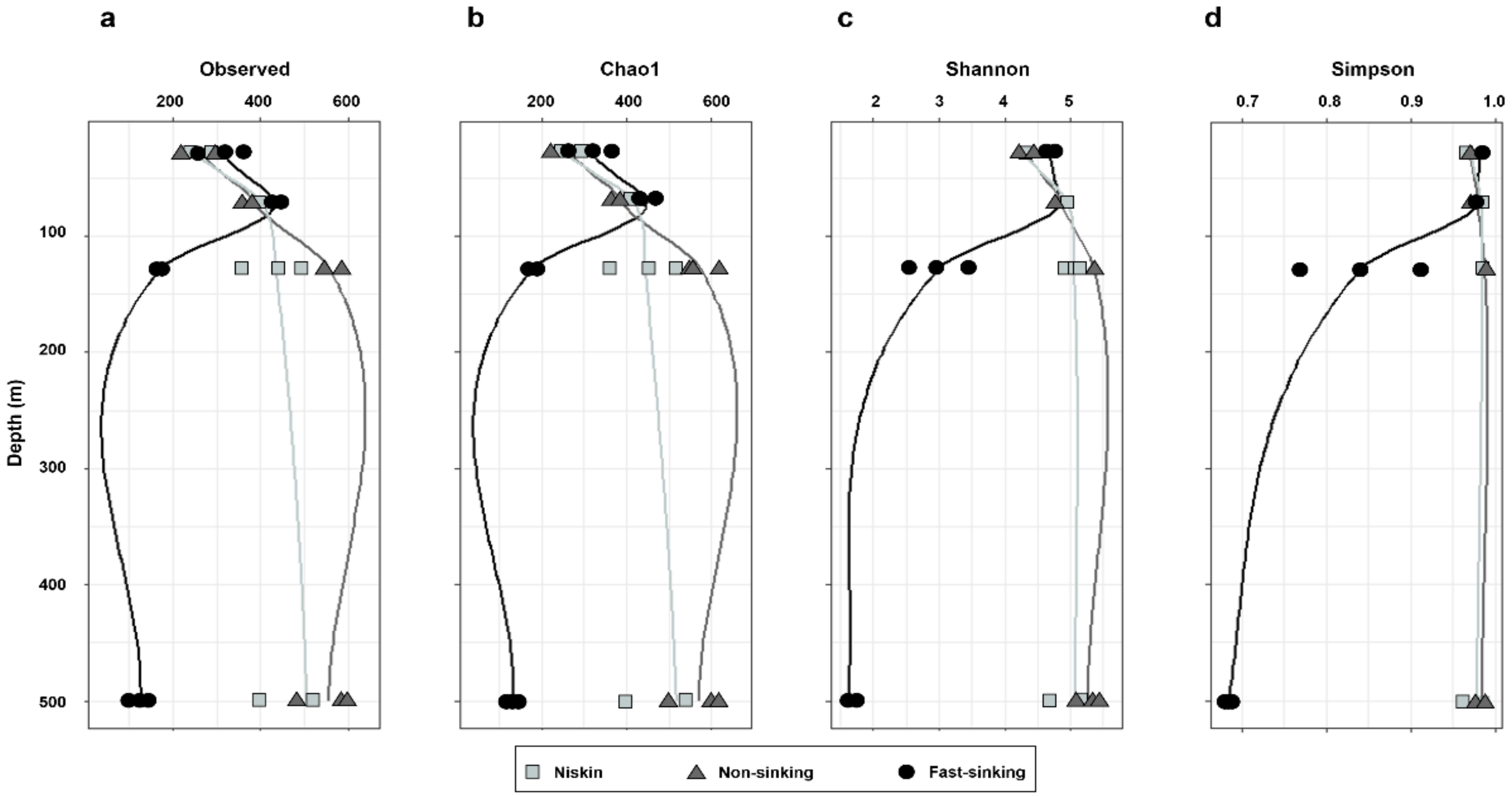

Figure S3: Depth variation of diversity indices (A) Observed species richness, (B) Chao1, (C) Shannon's and (D) Simpson's indices (see methods) in three different fractions: i) seawater sampled with Niskin bottles, ii) non-sinking and iii) fast-sinking prokaryotic fractions sampled with a marine snow catcher (MSC) at 4 water depths (28, 70, 128 and 500 m). Diversity indices were calculated from rarefied and normalized dataset (see methods).

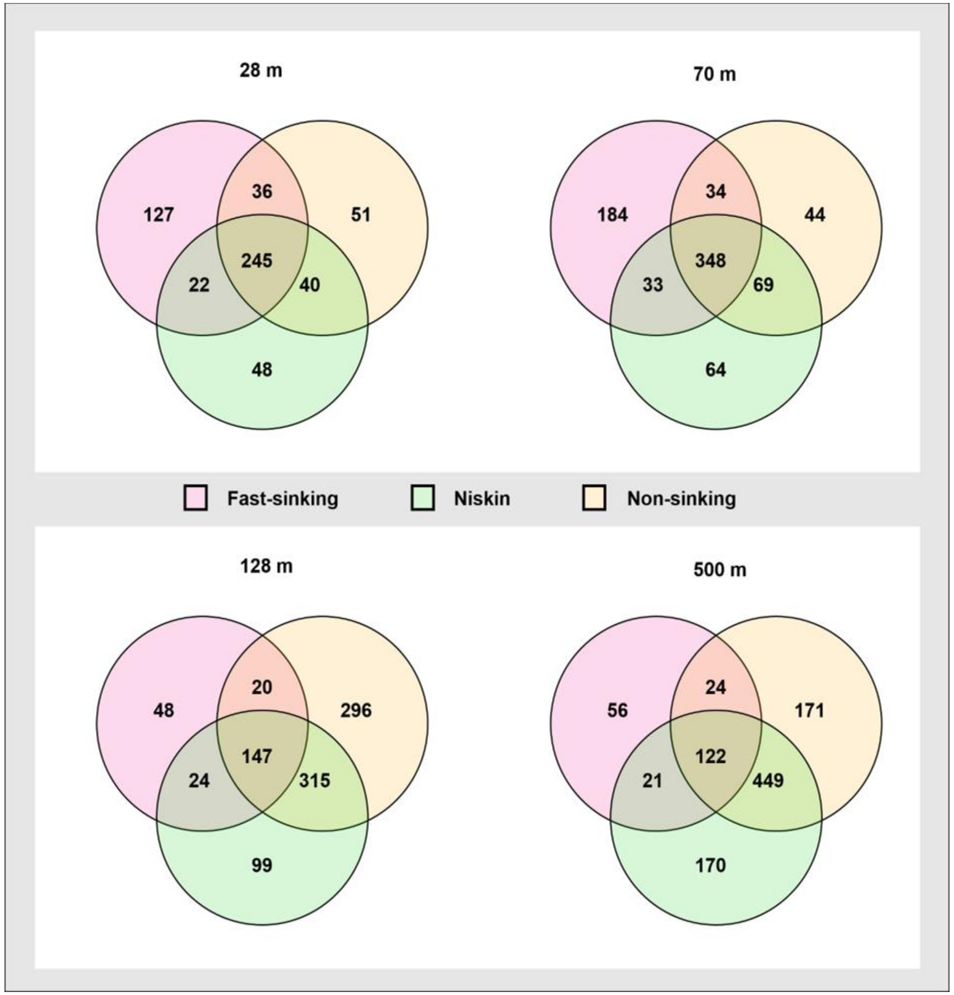

Figure S4: Venn diagram of OTU distribution of the normalized data between three different prokaryotic fractions: i) seawater sampled with Niskin bottles, ii) non-sinking and iii) fast-sinking prokaryotic fractions sampled with a marine snow catcher (MSC) at 4 water depths (28, 70, 128 and 500 m). Abundances of all specific and shared OTUs are shown.

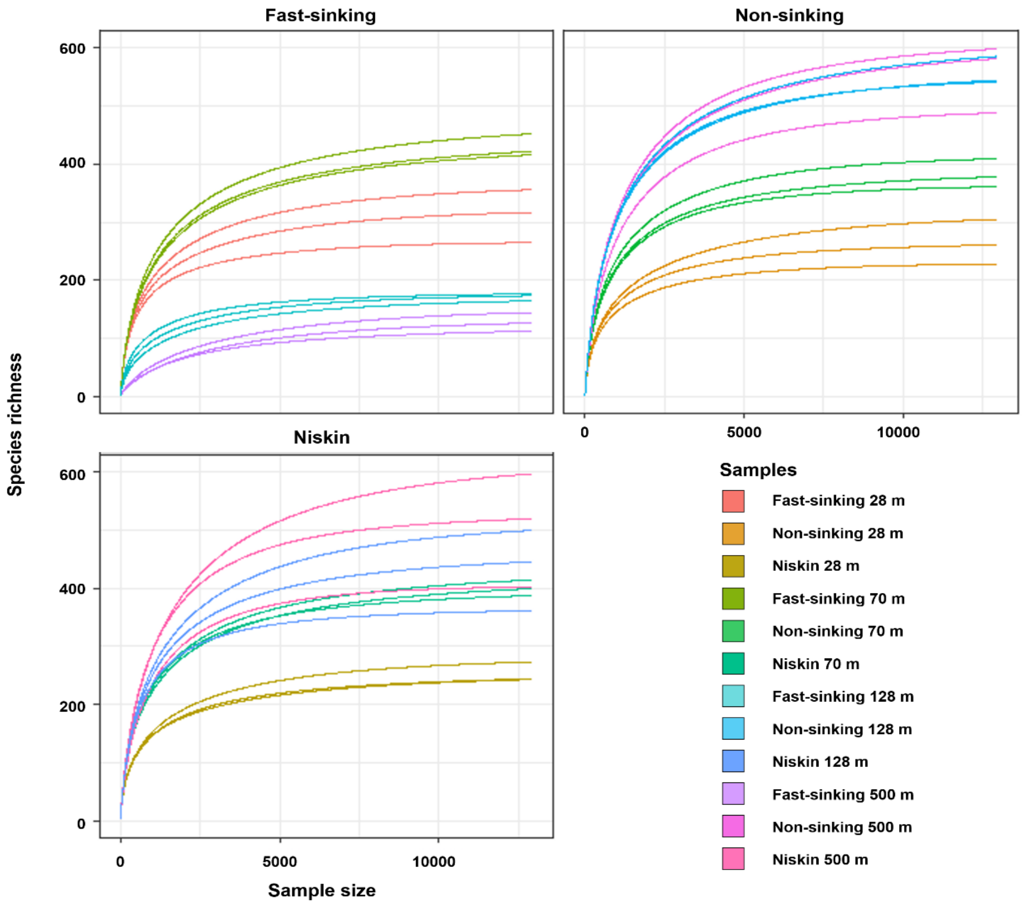

Figure S5: Rarefaction curves for individual samples from three different fractions: i) seawater sampled with Niskin bottles, ii) non-sinking and iii) fast-sinking prokaryotic fractions sampled with a marine snow catcher (MSC) at 4 water depths (28, 70, 128 and 500 m).

Table S1: PHP, cell concentration, cell-specific PHP and prokaryotic remineralization (PR or C loss) rates from three different fractions: i) seawater sampled with Niskin bottles, ii) non-sinking and iii) fast-sinking prokaryotic fractions sampled with a marine snow catcher (MSC) at 4 water depths (28, 70, 128 and 500 m). PR rates were obtained using a prokaryotic growth efficiency of 8%[3] for non-sinking samples. In contrast, PR rates for the fast-sinking samples were obtained applying a respiratory quotient RQ = 1 to respiration measurements published elsewhere[1] (see supplementary text).

Sample type	Depth	PHP (ng C L^{-1} h^{-1})	s.d.	Cell ml^{-1}	s.d.	PHPspec (ng C $cell^{-1}$ h^{-1})	s.d.	PR (ng C L^{-1} h^{-1})	s.d.
Fast sinking	28	0.211	0.014	2.27E+09	2.03E+09	1.29E-07	4.57E-08	5.316	4.902
Fast sinking	70	0.091	0.006	1.25E+09	7.40E+08	1.99E-07	2.67E-08	3.457	4.793
Fast sinking	128	0.031	0.032	3.03E+08	9.01E+07	4.00E-07	1.28E-08	1.566	1.419
Fast sinking	500	0.003	0.003	1.20E+08	6.39E+07	1.53E-07	1.66E-08	1.457	1.054
Non Sinking	28	111.755	10.99	2.76E+06	1.36E+06	4.05E-08	3.98E-09	1285.183	126.385
Non Sinking	70	11.917	0.364	8.50E+05	4.62E+05	1.40E-08	4.28E-10	137.046	4.186
Non Sinking	128	2.449	0.94	4.08E+05	1.63E+05	6.00E-09	2.30E-09	28.164	10.810
Non Sinking	500	1.162	0.186	2.67E+05	7.20E+04	4.35E-09	6.96E-10	13.363	2.139
Niskin	28	90.202	1.101	1.43E+06	8.04E+05	6.31E-08	7.74E-10	1037.323	12.662
Niskin	70	10.512	0.088	8.67E+05	NA	1.21E-08	1.47E-10	120.888	1.012
Niskin	128	2.465	0.066	3.59E+05	2.60E+04	6.86E-09	1.83E-10	28.348	0.759
Niskin	500	1.124	0.373	1.27E+05	8.69E+03	9.76E-09	2.93E-09	12.926	4.290

Table S2: Class level relative abundance (%) in three different fractions: i) seawater sampled with Niskin bottles, ii) non-sinking and iii) fast-sinking prokaryotic fractions sampled with a marine snow catcher (MSC) at 4 water depths (28, 70, 128 and 500 m).

	**Fast sinking**				**Non sinking**				**Niskin**			
**Class**	28 m	70 m	128 m	500 m	28 m	70 m	128 m	500 m	28 m	70 m	128 m	500 m
Gammaproteobacteria	18.229	28.848	71.165	94.917	18.159	15.599	23.227	22.082	16.811	14.049	22.800	28.593
Nitrososphaeria	0.103	2.167	0.250	0.139	0.075	11.283	12.137	13.115	0.015	5.775	11.306	11.496
Alphaproteobacteria	23.965	13.468	5.809	1.606	32.911	29.056	21.932	21.093	34.265	30.240	20.084	15.300
Oxyphotobacteria	14.479	9.234	1.305	0.540	17.866	6.987	1.441	1.493	13.751	5.955	3.451	0.770
Deltaproteobacteria	3.423	5.227	1.449	0.710	3.812	9.345	8.578	10.163	2.620	13.887	9.123	9.834
Thermoplasmata	0.396	0.211	0.003	0.005	1.320	1.979	2.190	2.839	1.529	2.339	1.889	1.995
Bacteroidia	33.655	31.437	17.179	1.508	18.947	10.925	8.001	9.540	23.600	9.823	5.924	3.088
Nitrospinia	0.015	0.265	0.039	0.013	0.000	0.893	1.840	2.131	0.008	0.399	1.951	1.585
Verrucomicrobiae	1.948	3.297	1.886	0.149	1.773	4.012	2.996	4.342	2.321	5.103	2.242	1.925
Acidimicrobiia	0.414	0.432	0.059	0.057	0.481	1.902	2.278	2.270	0.440	2.059	1.889	2.532
BD2-11_terrestrial_group	0.000	0.015	0.000	0.003	0.005	0.054	0.553	0.082	0.000	0.031	0.631	0.782
JG30-KF-CM66	0.000	0.010	0.000	0.000	0.000	0.095	0.533	0.311	0.000	0.028	0.255	0.273
Planctomycetacia	0.672	1.086	0.051	0.036	0.389	1.415	1.050	1.379	0.363	2.188	1.413	1.521
Dehalococcoidia	0.028	0.170	0.028	0.064	0.072	0.697	4.020	1.372	0.003	0.733	5.778	7.198
OM190	0.111	0.319	0.100	0.013	0.021	0.268	0.188	0.232	0.021	0.270	0.049	0.069
Bacilli	0.260	0.625	0.003	0.023	0.000	0.005	0.015	0.054	0.003	0.003	0.059	0.126
Dadabacteriia	0.028	0.015	0.000	0.000	0.062	0.054	0.064	0.067	0.036	0.098	0.033	0.003
Subgroup_6	0.000	0.000	0.000	0.000	0.000	0.000	0.206	0.000	0.000	0.000	0.492	0.824
BD7-11	0.131	0.098	0.018	0.026	0.044	0.062	0.023	0.049	0.044	0.046	0.041	0.028
Phycisphaerae	0.160	0.394	0.088	0.013	0.268	0.481	0.347	0.391	0.221	0.870	0.147	0.080
Lentisphaeria	0.051	0.183	0.203	0.067	0.000	0.033	0.036	0.098	0.000	0.036	0.152	0.170
Actinobacteria	0.389	0.651	0.003	0.057	0.013	0.021	0.008	0.021	0.018	0.010	0.018	0.069
Anaerolineae	0.000	0.008	0.000	0.000	0.000	0.000	0.072	0.008	0.000	0.000	0.080	0.281
Subgroup_26	0.000	0.000	0.000	0.000	0.000	0.000	0.041	0.000	0.000	0.000	0.093	0.100
Marine_Benthic_Group_A	0.010	0.000	0.000	0.000	0.000	0.000	0.036	0.000	0.000	0.000	0.144	0.165
MAST-3D	0.039	0.000	0.000	0.000	0.229	0.000	0.000	0.000	0.021	0.000	0.000	0.000
Pla3_lineage	0.000	0.000	0.000	0.010	0.000	0.000	0.023	0.000	0.000	0.000	0.051	0.095
**Others**	1.493	1.840	0.363	0.044	3.554	4.833	8.163	6.869	3.912	6.056	9.906	11.097

Table S3: Summary table of the various filters, denoised and chimera removals from the metabarcoding processing.

Sample type	Depth (m)	Imput (read #)	Filtered (read #)	Denoised (read #)	Merged (read #)	Nonchimeric (read #)	Final_retained (%)
Fast sinking	28	30805	26122	25571	25069	24931	80.93166694
Fast sinking	28	33131	27536	26935	26351	26220	79.14038212
Fast sinking	28	23553	19741	19274	18699	18622	79.0642381
Fast sinking	70	29674	24366	23761	23283	23190	78.14922154
Fast sinking	70	29608	24549	23949	23514	23423	79.11037557
Fast sinking	70	33914	27978	27454	27038	26926	79.39494014
Fast sinking	128	35978	29837	29172	28445	27006	75.06253822
Fast sinking	128	20551	17119	16645	16470	16386	79.73334631
Fast sinking	128	35940	30326	29920	29358	28231	78.55036171
Fast sinking	500	34550	29302	28868	28667	27678	80.10998553
Fast sinking	500	35567	29888	29491	29240	28108	79.02831276
Fast sinking	500	25586	21482	21061	20766	19962	78.01922927
Niskin	28	32425	27212	26437	25518	25276	77.95219738
Niskin	28	30443	25473	24868	24359	24198	79.486253
Niskin	28	28914	23900	23306	22732	22587	78.11786678
Niskin	70	36358	29964	28476	27156	27055	74.41278398
Niskin	70	38582	31973	30764	29519	29245	75.79959567
Niskin	70	29735	25037	24017	23185	23151	77.8577434
Niskin	128	29967	24581	23413	22339	21981	73.35068575
Niskin	128	17447	14478	13690	13091	12952	74.23625838
Niskin	128	43231	35861	34471	33099	32405	74.95778492
Niskin	500	21561	17780	16571	15109	15031	69.71383517
Niskin	500	37756	31195	29558	27403	27130	71.85612883
Niskin	500	26548	22005	20503	18533	18533	69.80940184
Non Sinking	28	30267	25080	24254	23453	23173	76.56193214
Non Sinking	28	35974	29976	29229	28653	28252	78.53449714
Non Sinking	28	24136	19874	19186	18498	18276	75.72091482
Non Sinking	70	31937	25956	24715	23595	23221	72.70877039
Non Sinking	70	32620	27204	26195	25081	24841	76.15266708
Non Sinking	70	30255	24972	24026	22975	22758	75.22062469
Non Sinking	128	29559	24313	22957	21183	21033	71.1559931
Non Sinking	128	31885	25956	24545	23059	22920	71.88333072
Non Sinking	128	36807	30531	29031	27213	26960	73.24693672
Non Sinking	500	26826	21859	20498	19358	19289	71.90412287
Non Sinking	500	33495	27674	26207	24311	24206	72.26750261
Non Sinking	500	36348	29755	28093	25121	25078	68.99416749

Table S4: Statistic tests between sample types for the different diversity indices.

		Observed		Chao1		Shannon		Simpson	
Krustal Wallis test	χ^2	32.37		32.19		32.48		28.51	
	p-value	0.0006653		0.0007096		0.0006391		0.002705	
Pairwise Wilcoxon		Fast-sinking	Non-sinking	Fast-sinking	Non-sinking	Fast-sinking	Non-sinking	Fast-sinking	Non-sinking
	Non-sinking	0.02	-	0.017	-	0.0044	-	0.067	-
	Niskin	0.036	0.488	0.031	0.478	0.0044	0.5137	0.025	0.755

Table S5: Diversity indices (see methods and supplementary text).

Sample type	Depth	Observed	Chao1	se.chao1	Shannon	Simpson
Fast sinking	28	317	321.5333	3.2152	4.6586	0.9810
Fast sinking	28	356	363.4412	4.3696	4.7740	0.9834
Fast sinking	28	265	265.7500	1.1092	4.6094	0.9816
Fast sinking	70	422	440.5000	8.0171	4.8138	0.9795
Fast sinking	70	416	430.1667	6.5151	4.8237	0.9824
Fast sinking	70	452	466.2500	6.2815	4.8408	0.9762
Fast sinking	128	174	176.8947	2.5828	2.9657	0.8411
Fast sinking	128	177	184.2000	6.4401	3.4405	0.9137
Fast sinking	128	165	169.5500	3.4511	2.5762	0.7689
Fast sinking	500	145	150.1000	3.4730	1.6812	0.6906
Fast sinking	500	127	136.0000	5.7053	1.5647	0.6791
Fast sinking	500	113	118.0556	3.7773	1.5896	0.6843
Niskin	28	273	279.3333	4.0845	4.3087	0.9715
Niskin	28	243	252.0000	5.5721	4.3589	0.9745
Niskin	28	244	249.4545	3.8322	4.2559	0.9693
Niskin	70	399	416.0323	7.8966	4.9189	0.9828
Niskin	70	414	433.8333	8.8765	4.9404	0.9829
Niskin	70	387	396.6250	5.5658	4.9711	0.9840
Niskin	128	445	456.1538	5.6059	5.0619	0.9850
Niskin	128	361	363.8947	2.5829	4.9731	0.9836
Niskin	128	499	513.3469	6.3810	5.1236	0.9854
Niskin	500	402	405.0000	2.7514	4.6960	0.9645
Niskin	500	596	614.2836	7.1056	5.2086	0.9831
Niskin	500	519	535.7143	8.5074	5.3316	0.9893
Non Sinking	28	261	265.8750	3.7683	4.3598	0.9730
Non Sinking	28	305	313.9032	5.0168	4.3780	0.9711
Non Sinking	28	228	228.5556	0.9403	4.2540	0.9692
Non Sinking	70	361	364.1429	2.6812	4.7870	0.9779
Non Sinking	70	409	412.8857	2.8430	4.9114	0.9807
Non Sinking	70	378	386.8846	5.1813	4.8338	0.9794
Non Sinking	128	541	548.6923	4.3630	5.3295	0.9880
Non Sinking	128	544	555.7333	5.6513	5.3537	0.9884
Non Sinking	128	585	618.1500	12.0885	5.3800	0.9886
Non Sinking	500	488	494.3250	3.8180	5.0503	0.9779
Non Sinking	500	581	603.1176	8.5532	5.3264	0.9870
Non Sinking	500	598	617.1333	7.9251	5.4261	0.9894

Supplementary References

1. Belcher A, Iversen M, Giering S, Riou V, Henson SA, Berline L, et al. Depth-resolved particle-associated microbial respiration in the northeast Atlantic. *Biogeosciences* 2016; **13**: 4927–4943.

2. Ploug H, Jorgensen BB. A net-jet flow system for mass transfer and microsensor studies of sinking aggregates. *Mar Ecol Prog Ser* 1999; **176**: 279–290.

3. Giering SLC, Sanders R, Lampitt RS, Anderson TR, Tamburini C, Boutrif M, et al. Reconciliation of the carbon budget in the ocean's twilight zone. *Nature* 2014; **507**: 480–483.

4. Riley J, Sanders R, Marsay C, Le Moigne FAC, Achterberg E, Poulton A. The relative contribution of fast and slow sinking particles to ocean carbon export. *Global Biogeochem Cycles* 2012; **26**.
